# Hypervirulent *Klebsiella pneumoniae* serotype K1 clinical isolates form robust biofilms at the air-liquid interface

**DOI:** 10.1371/journal.pone.0222628

**Published:** 2019-09-18

**Authors:** Meritxell Cubero, Sara Marti, Mª Ángeles Domínguez, Aida González-Díaz, Dàmaris Berbel, Carmen Ardanuy

**Affiliations:** 1 Department of Microbiology, Hospital Universitari de Bellvitge, Instituto de Investigación Biomédica de Bellvitge (IDIBELL), Barcelona, Spain; 2 Research Network for Respiratory Diseases (CIBERES), ISCIII, Madrid, Spain; 3 Spanish Network for Research in Infectious Diseases (REIPI), Barcelona, Spain; 4 Departamento de Patología y terapéutica experimental, Universitat de Barcelona, Barcelona, Spain; Universidad Nacional de la Plata, ARGENTINA

## Abstract

The prevalence of a new hypervirulent and hypermucoviscous *K*. *pneumoniae* phenotype (Hmv) is increasing worldwide, mainly linked to serotypes K1 and K2. Since capsular thickness can directly affect the capability to form biofilms, we aimed to evaluate the association between the Hmv phenotype with adhesion and biofilm formation in a collection of clinical *K*. *pneumoniae* isolates.

We selected 38 Hmv clinical isolates [15 serotype K1; 9 serotype K2; 3 non-K1/K2 (*rmpA*^+^); 11 non-K1/K2 (*rmpA*^-^)] and 7 non-Hmv clinical isolates. The Hmv phenotype was assessed through the mucoviscosity test. Serum resistance was determined by bacterial viability tests in pooled human serum. Adhesion was evaluated with the Biofilm Ring Test^®^, and biofilm formation was identified by crystal violet staining (Solid-Liquid, SLI-biofilm) or visual examination (Air-Liquid, ALI-biofilm).

This study linked for the first time the formation of robust ALI-biofilm plugs by *K*. *pneumoniae* to the capsular serotype K1, a group of hypervirulent strains which are generally highly susceptible to the antimicrobial agents. Among all the studied isolates, the capsular serotype K1 presented lower initial adhesion despite having the adhesins *mrkD* and *fimH* but higher ALI-biofilm formation than isolates with other capsular serotypes (K2 or non-K1/K2). This structure might confer increased resistance to a group of hypervirulent *K*. *pneumoniae* serotype K1.

## Introduction

*Klebsiella pneumoniae* is a human pathogen frequently causing nosocomial infections such as bloodstream and urinary tract infections, especially in intensive care units [1,2]. Its pathogenicity has been linked to the presence of virulence factors, including capsular antigens, lipopolysaccharide, adhesins and siderophores which are used for survival, adaptability and immune evasion during infection [2]. In addition, the ability to form a biofilm is considered an important colonization strategy among pathogenic *K*. *pneumoniae* strains. Biofilms are associated with enhanced resistance against antimicrobial agents, contributing to persistence of microbial infections, and favouring therapy failure [3].

Hypervirulent *K*. *pneumoniae* strains with hypermucoviscous phenotype (Hmv) have spread worldwide since 2004 when they were first identified in Asia. Hypermucoviscosity has been related with the capsular serotype K1, and in a lower proportion with the serotype K2, and has been linked to the presence of the *magA* (mucoviscosisty-associated gene A) and *rmpA* (regulator of mucoid phenotype A) genes [4,5].

The virulence plasmid pLVPK, which includes the *rmpA* gene, two siderophores (aerobactin and salmochelin) and a genomic island encoding yersiniabactin and colibactin, was described in hypervirulent serotype K1 *K*. *pneumoniae* strains [6]. This plasmid has high homology with the virulence plasmid pNTUH-K2044 described previously [7].

Hmv strains are commonly isolated from patients with a wide range of community acquired infections such as pyogenic liver abscess (PLA), pneumonia, endophthalmitis, meningitis, and necrotizing fasciitis [5]. Recently, our group has reported the prevalence (5.4%) of the Hmv phenotype among *K*. *pneumoniae* isolates from patients with bacteraemia in the Hospital Universitari de Bellvitge (HUB, Barcelona), together with genotypic differences regarding the presence of the *magA* and *rmpA* genes [5]. While it is known that Hmv capsule protects the cell from external factors (complement, anti-microbial peptides), it may also interfere with the function of other surface-located proteins such as type 1 fimbriae and nonfimbrial adhesins [8,9]. Since these membrane proteins are highly associated with biofilm formation, the presence of thick capsules in *K*. *pneumoniae* serotypes K1 and K2 could have a direct effect on their capability to form biofilms. Based on these premises, we aimed to test if there is an association between hypermucoviscosity and biofilm formation by *K*. *pneumoniae* that could be implicated in adaptation and bacterial survival. Although no correlation was observed between *K*. *pneumoniae* serotype K1 and SLI-biofilm formation, a significant association with formation of thick and viscous ALI-biofilm plugs or *floating* biofilm was showed.

## Materials and methods

### Characterization of bacterial strains

*K*. *pneumoniae* isolates with string test positive were considered hypermucoviscous (Hmv), while those isolates with either *magA* and/or *rmpA* were considered hypervirulent.

We analysed thirty-eight Hmv and seven non-Hmv *K*. *pneumoniae* isolates collected from blood samples in the Hospital Universitari de Bellvitge between 2007 and 2013 [5]. *K*. *pneumoniae* isolates were classified in four groups depending on the hypermucoviscous phenotype and presence/absence of *magA* and/or *rmpA*: Hmv *magA*(+)/*rmpA*(+) [group1], Hmv *magA*(-)/*rmpA*(+) [group2], Hmv *magA*(-)/*rmpA*(-) [group3], and non-Hmv *magA*(-)/*rmpA*(-) [group4]. (S1 Table)

#### Bacterial phenotype and mucoviscosity assay

The Hmv phenotype was identified by the string test, and the hypermucoviscosity related genes (*magA* and *rmpA*) were detected by PCR [4,10]. Hypermucoviscosity was quantified by comparing the degree of bacterial compactness in a pellet after centrifugation following previously described methodologies with some modifications [11,12]. Strains were grown in 1mL of Luria Bertani (LB) broth at 37°C overnight with shaking. The samples were centrifuged at 1,000×g for 5 min and the absorbance was measured at 600 nm (OD_600_).

#### Bacterial genotyping and virulence factors

All isolates were genotyped by multilocus sequence typing (MLST). K-antigen serotypes (capsular polysaccharide typing) were identified by sequencing the specific *wzi* allele as previously described [5]; all strains included in this study have a *wzi* allele. Virulence associated genes were identified by PCR using primers for the detection of siderophores (*iroN*, salmochelin; *ybtS*, yersiniabactin; *entE* and *entB*, enterobactin; *iucA*, aerobactin; *clbA*, colibactin), ferric uptake system (*kfu*), adhesins (*mrkD*, *fimH*), and an activator of the allantoin regulon (*allS*) [2,13–15].

#### Serum resistance assay

The ability of the isolates to resist killing by human serum was determined using a modified version of a previously established methodology [10,16,17]. Bacteria were grown in BHI at 37°C with shaking to an OD_600_ of 0.5–0.6, washed and diluted to an OD_600_ of 0.1 in PBS-Hanks’ Balanced Salt solution (HBSS). Samples were further diluted 1,000-fold in PBS-HBSS. The diluted suspensions were mixed at a 1:3 vol/vol with normal human serum (NHS) (Sigma-Aldrich) or heat inactivated normal human serum (HI-NHS), and incubated at 37°C for two hours. The number of viable bacteria was determined by plating serial dilutions.

### Bacterial adhesion assessment

Adherence to the solid surface was measured using a paramagnetic beads-mediated agglutination assay (BioFilm Ring Test^®^, Biofilm Control, France), following previously described methodology with some modifications [18,19]. Bacterial suspensions were mixed with magnetic beads and incubated at 37°C for 5h. The biofilm index (BFI) was adjusted by the BioFilm Ring Test^®^ software and was inversely proportional to the number of adherent bacteria. BFI values >7 correspond to a total lack of bacterial adherence, while only values of <5 were associated with different degrees of bacterial adhesion. The adhesion experiments were performed by triplicate and on two different days.

### Solid-liquid biofilm formation assay (SLI biofilm)

The ability of bacteria to form a biofilm was assessed on 96-well plates with crystal violet staining following the methodology previously described [17,18]. Overnight bacterial cultures were diluted to an OD_600_ of 0.01 in Brain Heart Infusion (BHI) broth and incubated at 37°C for 24h. After incubation, the OD_600_ was determined to assess bacterial growth. Biofilm was stained with 0.5% crystal violet at room temperature, washed and dissolved with 90% ethanol. Biofilm quantification was obtained by measuring the absorbance at 570 nm (A_570_). Biofilm values were obtained by calculating the mean of the absorbance for at least three independent experiments and compared to negative controls (BHI). Biofilm formation was considered positive when the readings were at least 3 times greater than the negative control.

### Air-liquid biofilm formation assay (ALI biofilm)

ALI biofilm formation was performed in 12 mm diameter polystyrene tubes as previously described with small modifications [20]. A single bacterial colony was resuspended into 2 mL BHI broth, vortexed thoroughly and incubated for 72h at 37°C. ALI biofilm-producing strains were identified by visual examination of the air-liquid interface; the strains were considered positive when a thick pellicle was covering the liquid surface.

### Statistical analysis

Statistical analyses were performed with the GraphPad Prism version 5 software, where *P* value less than 0.05 was considered statistically significant. Means ± standard errors of the means of three independent replicates are depicted. One-way analysis of variance with the Newman-Keuls multiple-comparison *post hoc* test was used for statistical analysis.

### Ethics statement

Written informed consent was not considered necessary for the study because it was a retrospective analysis of *K*. *pneumoniae* isolates kept in the Microbiology department biobank. Patients data were anonymized for analysis and handling. Confidential information from individual patients was protected according to national guidance.

## Results

### Characterization of bacterial strains

**Fig 1:**
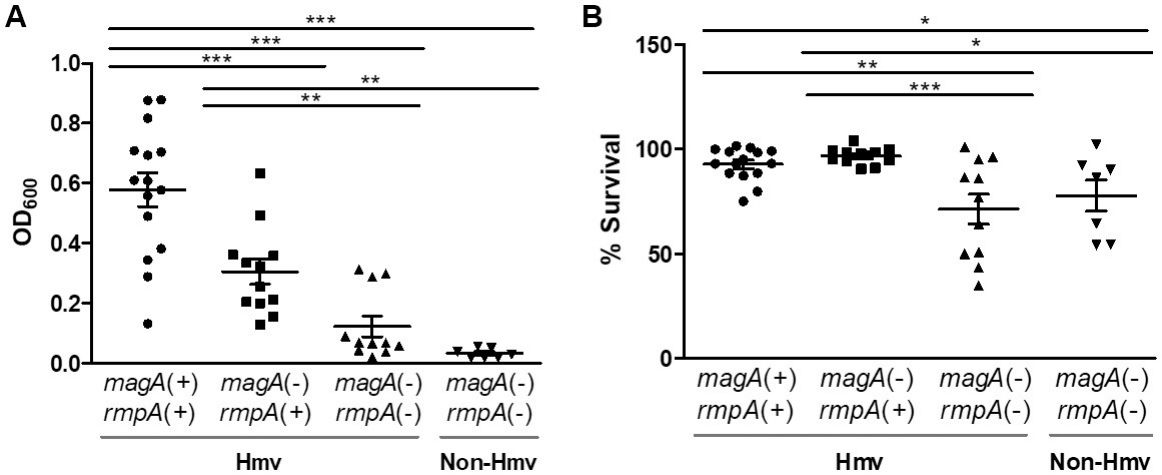
Mucoviscosity and resistance to human serum linked to the Hmv-associated genes (*magA* and/or *rmpA*). **A.** Hypermucoviscosity levels were determined as the OD_600_ of the supernatant obtained after centrifugation of an overnight culture at 1,000×g for 5 minutes. **B.** Survival of *K*. *pneumoniae* strains in 75% NHS compared to 75% HI-NHS. Means ± standard errors of the means of three independent replicates are depicted. One-way analysis of variance with the Newman-Keuls multiple-comparison *post hoc* test was used for statistical analysis (* *P*<0.05; ** *P*<0.01; *** *P*<0.001).

The presence of a mucoid capsule hampers pellet formation after culture centrifugation, facilitating the quantification of different degrees of bacterial mucoviscosity. As expected, Hmv *K*. *pneumoniae* strains presented a less compact pellet after centrifugation than non-Hmv strains and these differences correlated with the number of hypermucoviscosity genes identified and string test classification (Fig 1A, Table 1). Likewise, these Hmv strains presenting one or two Hmv-associated genes (*magA* and/or *rmpA*) also had the highest serum resistance levels (Fig 1B) and number of virulence determinants (Table 1).

**Table 1:** Clinical isolates selected for the present study. Distribution of virulence factors determined in *K*. *pneumoniae* in different groups based on the presence or absence of *magA* and *rmpA* genes.

Phenotype	*magA*	*rmpA*	K-Serotype	ST	No. Strains	Siderophore genes ^a^				Iron uptake system	Allantoin regulation	Adhesins	
						*iroN*	*ybtS*	*iucA*	*clbA*	*kfu*	*allS*	*mrkD*	*fimH*
Hmv [String test (+)]	+	+	K1	23	12	12	12	12	12	12	12	12	12
					1	1	0	1	0	1	0	1	1
					2	0	0	1	0	0	0	0	0
	-	+	K2	25	1	1	0	1	0	0	0	1	0
				65	1	0	0	0	0	0	0	0	0
				86	3	3	1	3	0	0	0	3	3
				380	3	3	3	3	3	3	0	3	3
				493	1	1	1	1	0	1	0	1	1
			Non K1/K2	5 ^b^	3	3	3	3	0	0	0	3	3
	-	-	Non K1/K2	17, 37, 321, 622, 719, 895(2)	7	0	0	0	0	0	0	7	7
				45, 152	2	0	2	0	0	0	0	2	2
				460	1	0	0	0	0	0	0	0	0
				1035	1	0	0	1	0	1	1	0	0
Non-Hmv [String test (-)]	-	-	Non K1/K2	35	2	1	1	0	0	1	0	2	2
				17, 104, 214, 465, 711	5	0	0	0	0	0	0	5	5

Results are expressed as number of positive strains.

^a^ The siderophore genes *entE* and *entB* were present in all the studied strains

^b^ This strain was previously described as ST1013 (SLV of ST5)

In addition to Hmv-associated genes, several virulence factors were identified in our strains suggesting the presence of a virulence plasmid. Among the siderophore genes, *entE* and *entB* were present in all the studied strains, while *clbA* was only detected in serotype K1 (12/15, 80%) and serotype K2 (3/9, 33%) strains. In addition, yersiniabactin (*ybtS*) was mostly identified in serotype K1 (12/15, 80%) and serotype K2 (5/9, 55%) strains, although it was also detected in non-K1/K2 Hmv or non-Hmv strains (6/21, 29%) demonstrating that did not belong strictly to the hypervirulent strains.

In addition, the *allS* gene, linked to allantoin metabolism, was only present in serotype K1 strains (12/15, 80%) and in one non-K1/K2 Hmv *K*. *pneumoniae* strain (ST1035). With this exception, Hmv strains lacking *magA* and *rmpA* were genotypically like the control non-Hmv strains with very low identification of siderophores, iron uptake systems and allantoine metabolism, while being positive for the fimbrial adhesin-encoding *mrkD* and *fimH* genes like most of the studied strains.

### Bacterial adhesion

Bacterial adhesion was determined using a magnetic beads-mediated agglutination assay (BioFilm Ring Test^®^). The biofilm index (BFI) obtained was inversely proportional to adhesion; only BFI values <5 were associated with bacterial adherence to the plate.

**Fig 2:**
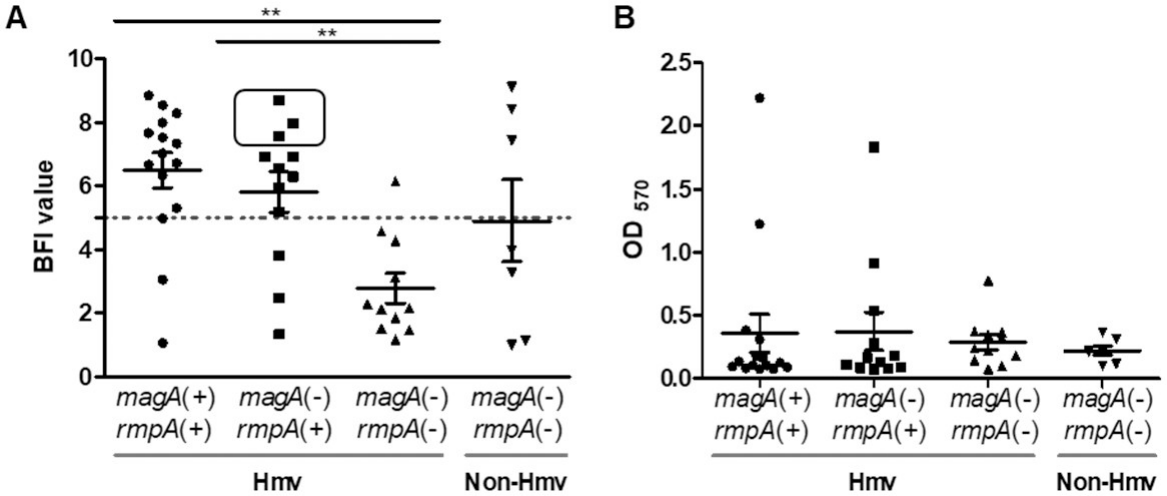
Adhesion and biofilm formation in *K*. *pneumoniae* isolates. **A.** Early-stage adhesion to a solid surface determined by the BioFilm ring test after 5h of static growth at 37°C. Dotted line represents the cutoff for adhesion (BFI = 5), with values of <5 representing high levels of adhesion to the surface. Square shows the three non-K1/K2 Hmv *K*. *pneumoniae* ST5 strains. **B.** Biofilm formation measured by crystal violet light absorbance at 570 nm (OD_570_). Means ± standard errors of the means of three independent replicates are depicted. One-way analysis of variance with the Newman-Keuls multiple-comparison *post hoc* test was used for statistical analysis. (* *P*<0.05; ** *P*<0.01; *** *P*<0.001).

After 5 hours of incubation nearly half of the strains (20/45, 44.5%) had adhered to the solid surface and this adhesion was mainly associated with the group of Hmv *K*. *pneumoniae* strains without *magA* and *rmpA* genes (Fig 2A). This early-stage *in vitro* adhesion to the solid surface was low for the Hmv phenotype associated with the presence of the hypermucoviscosity genes, including three non-K1/K2-ST5 strains, which presented the lowest adhesion values among *magA*(-)*/rmpA*(+) strains. Only five of the 27 (19%) Hmv strains harbouring *magA* and/or *rmpA* genes presented a strong early-stage adhesion to the solid surface (BFI values <5), in contrast with 10 out of the 11 (91%) Hmv *K*. *pneumoniae* strains lacking these Hmv-associated genes.

### Biofilm formation

The isolates were subjected to the detection of solid-liquid (SLI) biofilm formation by the static microtiter plate method and crystal violet staining. As shown in Fig 2B, most strains formed a weak biofilm (40 out of 45 strains, 89%), while only five strains produced a strong biofilm (11%). Biofilm formation did not differ among groups and it was not associated with any of the other tested variables (presence of Hmv-associated genes, hypermucoviscosity rate, bacterial adhesion or serum resistance), suggesting that biofilm formation could be a specific feature of each strains. Nevertheless, variability in the crystal violet quantification was observed in some Hmv strains due to bacterial attachment and physical contact between the bacteria and the wall of the tube. The diversity in the biofilm growth indicates the production of a second biofilm structure, which assists the early phase of colonization and subsequent attach irreversibly to surfaces.

**Fig 3:**
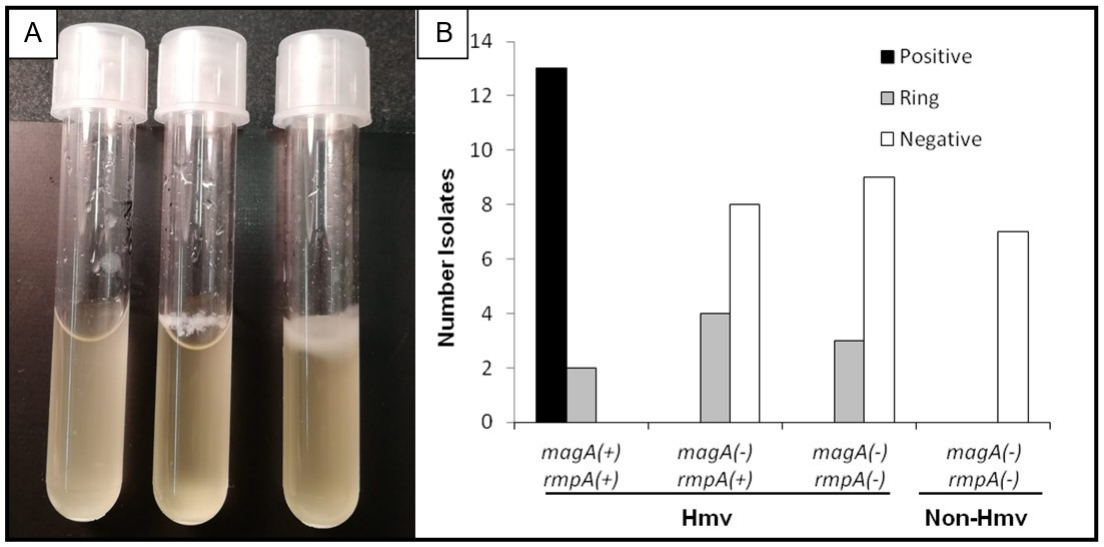
Biofilm at the interface air-liquid (ALI-biofilm). **A.** Representative sample of ALI-biofilm. The positive strain (right tube) forms a thick, while in the negative strain (left tube) no pellicle has been detected. The ring structure attaches to the wall of the tube (central tube). **B.** Distribution of the isolates with ability to form ALI-biofilm with respect to the presence of hypermucoviscosity genes. Positive: Pellicle covering all liquid surface; Ring: Pellicle forming on the walls of the tube but not covering all liquid surface; Negative: No biofilm formation.

Therefore, ALI biofilm formed at the air-liquid interface was studied by growing bacterial cultures without shaking in polystyrene tubes. ALI biofilm formation was considered positive when a consistent layer of biofilm was generated on the top of the liquid surface. As observed in Fig 3A, the ALI biofilm structure formed by some Hmv strains was so thick that it could support the weight of the liquid media after reversing the tube. This floating biofilm formation was associated with the presence of the hypermucoviscosity genes (Fig 3B). Only two out of 15 serotype K1 *K*. *pneumoniae* strains presented an incomplete ALI biofilm structure that was not completely covering the surface of the liquid media (ring structure attached to the wall of the tube). Those Hmv strains had been recovered from patients with intravenous catheter-related bacteraemia and were positive for all the studied virulence genes. Conversely, *K*. *pneumoniae* strains presenting the *rmpA* gene alone or no Hmv genes were mostly negative for ALI biofilm formation, despite forming occasionally a biofilm ring at the liquid-air interface.

## Discussion

*K*. *pneumoniae* is a commensal and opportunistic pathogen found in the nasopharynx and gastrointestinal tract of healthy individuals that causes a wide variety of infections particularly among patients with underlying diseases. While most infections in Western countries are due to “classic” *K*. *pneumoniae* strains, hypervirulent and hypermucoviscous lineages widely spread in Asian countries are also emerging around the world [21]. Moreover, and most alarming, recent reports showed the isolation of hypervirulent and multidrug resistant strains in these countries [6]. We recently screened all the Hmv *K*. *pneumoniae* isolates obtained from bacteraemia between 2007 and 2013 [5]. Although this Hmv phenotype makes bacteria easy to identify, little is known about the genotypic characteristics and virulence factors associated with this hypervirulent variants.

The ability to resist to human serum is an important virulence factor used to evade the host defence that has been correlated with the onset of the infection and the severity of the symptoms [2]. There are two hypotheses about the mechanisms underlying bacterial serum resistance and the compounds implicated in this resistance. In *K*. *pneumoniae* serotype K1, the capsular polysaccharide (CPS) may mask the lipopolysaccharide (LPS) changing the surface structure to avoid complement activation, while in the serotype K2, long O-polysaccharide side chains of the LPS may be exposed on the surface, producing complement activation without cell damage [22]. In K1 strains, *magA* gene has also been described located very close to *gmd* (GDP-mannose 4,6–dehydratase) and *wcaG* (GDP-keto-6-deoxymannose 3,5 epimerase/4-reductase) genes [23]. Both genes were implicated in the conversion of mannose into fucose, a sugar involved in the evasion of the immune response, such as evading phagocytosis masking K1 capsular epitope.

In other way, we observed higher serum resistance levels in the strains from serotypes K1, K2 and three non-K1/K2 with ST5 presenting the Hypervirulent-associated genes (*magA* and/or *rmpA*) [5]. As a matter of fact, this ST5, recently described by our group [5], presented a virulence phenotype and genotype comparable to the serotypes K1 or K2.

Consistent with these results, most of the studied virulence determinants were also detected among K1-ST23 strains [*magA*(+) and *rmpA*(+)], and in a lower degree, by non-K1 strains harbouring the *rmpA* gene (K2-ST86 and K2-ST380, and the non-K1/K2-ST5). Despite *magA* gene is extensively described exclusively by K1 *K*. *pneumoniae* strains, it was renamed as *wzy*_K1 (serotype K1 *wzy* allele: AB355924) [24].

Recently, Struve *et al* [6], identified a virulence plasmid highly homologous to pNTUH-K2044 [7], containing the regulator of mucoid phenotype (*rmpA*) and two siderophores (aerobactin and salmochelin), and also a genomic island with gene clusters encoding yersiniabactin and colibactin in hypervirulent serotype K1 *K*. *pneumoniae* strains. A large virulence plasmid with these virulence factors was first described in a *K*. *pneumoniae* strain K2 [6,25]. Our results show that most of the *rmpA* positive strains (24/27, 89%), including K1, K2 and non-K1/K2-ST5, also contained the siderophores aerobactin and salmochelin, suggesting the presence of this virulence plasmid. In addition, genes encoding for yersiniabactin (*ybtS*) and colibactin (*clb*) were identified in most serotype K1 strains, but also in the three serotype K2-ST380 strains, in agreement with previous reports linking this clonal complex with hypervirulence [26].

Previous studies on serotypes K1 and K2 *K*. *pneumoniae* have already identified these hypervirulent-associated genes. However, little is known about the ability of these strains to adhere and form a biofilm. For that reason, we analyzed these features in a collection of Hmv *K*. *pneumoniae* isolates with and without *magA* and/or *rmpA* genes.

Adhesins play an essential role in the initial surface adhesion required to form a biofilm and in the posterior biofilm development as they promote cellular contact. Most *K*. *pneumoniae* strains express either type 1 or type 3 fimbriae and their expression in biofilm vary depending on the experimental conditions [27]. The bacterial adherence capabilities might be beneficial for the pathogen to form a biofilm, contributing, for instance, to the chronicity of infections [28]. Our results show a meaningful inverse relationship between initial adhesion to a solid surface and the presence of the hypermucoviscosity genes. This relationship was present in Hmv strains from serotypes K1-ST23, K2 (ST86 and ST380), and non-K1/K2-ST5, despite having the *mrkD* and *fimH* genes that codify for type 3 and type 1 fimbriae respectively [29]. Recently, Guo *et al* [30] reported that *mrkD* was only found among serotype K2 Hmv *K*. *pneumoniae*, being not identified among serotype K1 and non-Hmv strains [30]. By contrast, our results show that this adhesin is widely distributed among *K*. *pneumoniae* isolates.

This low adhesion suggests that the hypermucoviscosity may interfere with the bacterial ability to adhere to a solid surface *in vitro* blocking the biofilm formation. It has been shown that in Gram negative bacteria some proteinaceous bacterial adhesins can be hidden by the presence of a capsule [8]. Therefore, Hmv *K*. *pneumoniae* isolates carrying the cps loci for K1 and K2 capsule would mask not only the LPS, making the strains resistant to human serum, but also other proteins from the outer membrane such as fimbriae. Additionally, the organization of the *K*. *pneumoniae* capsule has already been implicated with biofilm formation, suggesting that capsular fluidity would be essential for optimal bacterial adhesion to the surface [8,31]. Some of the limitations of this manuscript could be the lack of information of the capsule composition.

Despite the low-level adhesion observed *in vitro*, we analyzed the capacity of these *K*. *pneumoniae* strains to form a biofilm *in vitro*. In a previous study, higher levels of biofilm were observed among PLA-associated *K*. *pneumoniae* [11], in contrast with our results in which no significant differences in biofilm formation have been detected between PLA-associated *K*. *pneumoniae* and other *K*. *pneumoniae* non-tissue-invasive isolates. In fact, regarding biofilm formation, no differences were observed among the four studied groups based on the Hmv phenotype and the presence or absence of the *magA* and *rmpA* genes. This variability suggests either that these two genes do not affect biofilm formation *in vitro* or, these strains have a reduced capability to form a biofilm due to the inability of the adhesins to adhere to the surface as discussed before [31]. Previous studies proposed the involvement of several factors as capsular fluidity or biofilm-related genes (*treC* and *sugE*) which affect biofilm formation, bacterial mucoviscosity or CPS production [11,31]. Although other authors have already observed a great variability on biofilm formation among tested carbapenem-resistant *K*. *pneumoniae* strains [16], we had no possibility to demonstrate this fact due to the absence of carbapenem-resistant isolates into our strains collection.

Despite the lack of association between the Hmv phenotype and biofilm formation, we observed some variability in biofilm quantification by crystal violet in Hmv *K*. *pneumoniae* K1 isolates which brought us to study the biofilm formed at the air-liquid interface (ALI-biofilm or pellicle). This “floating” biofilm represents a good ecological niche because it provides nutrients from the liquid media but also oxygen from the air and it has already been described in pathogenic bacteria such as *Acinetobacter baumannii*, *Pseudomonas aeruginosa*, *Escherichia coli*, *Salmonella enterica* or *Vibrio cholerae* [32]. We observed a strong correlation between ALI-biofilm formation and the presence of the *magA* gene (capsular serotype K1), with only two isolates producing a wicker pellicle that was not always able to cover the whole surface of the liquid media. In *E*. *coli*, floating biofilm formation has been associated with the presence of curli, flagella, Type 1 pili, siderophores, exopolysaccharide and the sensor kinase QseC [33], and recently, the presence of polar flagella has also been reported in one *K*. *pneumoniae* isolate [34]. Despite that, to our knowledge, this is the first time hypervirulent-hypermucoviscous *K*. *pneumoniae* serotype K1 are associated with the formation of this floating biofilm structure.

Concluding, we linked for the first time the formation of ALI-biofilm in *K*. *pneumoniae* to the capsular serotype K1. Among all the studied isolates, the capsular serotype K1 presented lower initial adhesion despite having the adhesins *mrkD* and *fimH* but higher ALI-biofilm formation than isolates with other capsular serotypes (K2 or non-K1/K2). This structure might confer increased resistance to a group of hypervirulent *K*. *pneumoniae* serotype K1.

## Supporting information

S1 Table Demographic, antibiotic MIC and genetic metadata. AMI, amikacin; AMC, amoxicillin/ac. clavulanic; AMP, ampicillin; CFP, cefepime; CFU, cefuroxime; CFZ, cefazoline; CTX, cefotaxime; CTZ, ceftazidime; AZT, aztreonam; SXT, trimethoprim-sulfamethoxazole; CIP, ciprofloxacin; OFX, ofloxacin; TOB, tobramycin; PPC, piperacillin; TIC, ticarcillin; PTZ, piperacillin-tazobactam.
(XLSX)
